# Cerebral hemodynamic response to upright position in acute ischemic stroke

**DOI:** 10.3389/fneur.2024.1392773

**Published:** 2024-07-11

**Authors:** Lilian B. Carvalho, Tina Kaffenberger, Brian Chambers, Karen Borschmann, Christopher Levi, Leonid Churilov, Vincent Thijs, Julie Bernhardt

**Affiliations:** ^1^Stroke Theme, Florey Institute of Neuroscience and Mental Health, University of Melbourne, Heidelberg, VIC, Australia; ^2^Neurology Department, Austin Health, Melbourne, VIC, Australia; ^3^Allied Health, St Vincent's Hospital, Melbourne, VIC, Australia; ^4^John Hunter Hospital, University of Newcastle, Newcastle, NSW, Australia; ^5^Department of Medicine (Austin Health) and Melbourne Brain Centre at Royal Melbourne Hospital, Melbourne Medical School, University of Melbourne, Melbourne, VIC, Australia

**Keywords:** ischemic stroke, early mobilization, upright position, sitting, standing, transcranial Doppler, hemodynamics

## Abstract

**Introduction:**

Concerns exist that a potential mechanism for harm from upright activity (sitting, standing, and walking) early after an acute ischaemic stroke could be the reduction of cerebral perfusion during this critical phase. We aimed to estimate the effects of upright positions (sitting and standing) on cerebral hemodynamics within 48 h and later, 3–7 days post-stroke, in patients with strokes with and without occlusive disease and in controls.

**Methods:**

We investigated MCAv using transcranial Doppler in 0° head position, then at 30°, 70°, 90° sitting, and 90° standing, at <48 h post-stroke, and later at 3–7 days post-stroke. Mixed-effect linear regression modeling was used to estimate differences in MCAv between the 0° and other positions and to compare MCAv changes across groups.

**Results:**

A total of 42 stroke participants (anterior and posterior circulation) (13 with occlusive disease, 29 without) and 22 controls were recruited. Affected hemisphere MCAv decreased in strokes with occlusive disease (<48 h post-stroke): from 0° to 90° sitting (−9.9 cm/s, 95% CI[−16.4, −3.4]) and from 0° to 90° standing (−7.1 cm/s, 95%CI[−14.3, −0.01]). Affected hemisphere MCAv also decreased in strokes without occlusive disease: from 0° to 90° sitting (−3.3 cm/s, 95%CI[−5.6, −1.1]) and from 0° to 90° standing (−3.6 cm/s, 95%CI [−5.9, −1.3]) (*p*-value interaction stroke with vs. without occlusive disease = 0.07). A decrease in MCAv when upright was also observed in controls: from 0° to 90° sitting (−3.8 cm/s, 95%CI[−6.0, −1.63]) and from 0° to 90° standing (−3 cm/s, 95%CI[−5.2, −0.81]) (*p*-value interaction stroke vs. controls = 0.85). Subgroup analysis of anterior circulation stroke showed similar patterns of change in MCAv in the affected hemisphere, with a significant interaction between those with occlusive disease (*n* = 11) and those without (*n* = 26) (*p* = 0.02). Changes in MCAv from 0° to upright at <48 h post-stroke were similar to 3–7 days. No association between changes in MCAv at <48 h and the 30-day modified Rankin Scale was found.

**Discussion:**

Moving to more upright positions <2 days post-stroke does reduce MCAv in the affected hemisphere; however, these changes were not significantly different for stroke participants (anterior and posterior circulation) with and without occlusive disease, nor for controls. The decrease in MCAv in anterior circulation stroke with occlusive disease significantly differed from without occlusive disease. However, the sample size was small, and more research is warranted to confirm these findings.

## 1 Introduction

The effects of upright positions (sitting and standing) on cerebral hemodynamics in acute ischemic stroke are not well understood. In theory, lying flat could increase cerebral perfusion, and upright activities could worsen perfusion, further damaging viable tissue. However, there is not enough evidence to support these statements and, therefore, to inform the development of head positioning protocols early post-stroke (1). In our recent systematic review of head positioning in ischemic stroke, which incorporated varied imaging techniques, cerebral blood flow (CBF) parameters were greater when lying flat compared to elevated head positions in most studies (2). However, most studies were underpowered, the reported changes were insignificant, and they did not assess longer-term outcomes. Our review also highlighted the lack of studies that examined changes in CBF parameters in upright positions in acute stroke. Sitting was assessed in only three studies: two within a week of stroke (3, 4) and one many years post-stroke (5). Standing was only assessed years post-stroke (5). To date, no studies have included all the postural changes from lying flat, head-up tilt, sitting, and standing to examine changes in CBF parameters in people with stroke compared to controls.

The international AVERT trial, which showed that stroke patients (ischemic and hemorrhagic) treated with very early, intensive, upright mobilization started < 24 h (VEM) after stroke had worse 3-month outcomes, raised concerns about the possible harms of early upright activity (6). A potential mechanism for harm in those with ischemic stroke is that VEM-associated upright activity (sitting, standing, and walking) worsens cerebral perfusion within penumbral tissue. A number of potential mechanisms may contribute to reduced cerebral perfusion during early upright activity. First, gravitational forces may act and lead to orthostatic reductions in cerebral perfusion as the head rises above the heart. While these changes are immediately controlled by cerebral autoregulation in healthy individuals, (7, 8) cerebral autoregulation is commonly impaired in acute stroke (9–11) and in people with occlusive disease (12, 13). Therefore, in upright postures, a defective cerebral autoregulation system may be unable to maintain sufficient blood flow to the ischaemic tissue, potentially promoting further damage. Second, occlusive disease, e.g., large vessel occlusion or severe arterial stenosis, often present in people with acute ischemic stroke, could hinder CBF downstream and further reduce CBF to the ischaemic penumbra. This could happen due to a reduction in the arterial lumen caused by the presence of a thrombus or atherosclerotic plaque in a large vessel supplying the affected ischaemic area. Moreover, cerebral autoregulation is known to be more affected in people with these conditions (12–14). Unfortunately, occlusive disease status was not recorded in AVERT, nor were there studies in our review examining CBF in upright positions in this population (2). Therefore, the specific effects of early upright activity on those with and without occlusive disease are unknown.

We aimed to investigate the effects of changes in head position from lying flat (0°) to 30°, 70°, 90° sitting, and 90° standing on middle cerebral artery mean velocity (MCAv) using transcranial Doppler (TCD) in people with ischemic stroke < 48 h of onset, with or without occlusive disease, and in controls. Our primary hypothesis was that a reduction in MCAv from lying flat (0°) to upright positions (sitting or standing) on the affected hemisphere would be greater in stroke participants with occlusive disease than those without. The secondary aim was to investigate whether changes in MCAv from lying flat (0°) to upright positions in the affected hemisphere in patients with ischaemic stroke would differ from those in the unaffected hemisphere and controls. We hypothesized that there would be a greater reduction in MCAv at upright positions in the affected hemisphere compared to the unaffected hemisphere in patients with stroke, and these changes would also be greater compared to controls. We also aimed to investigate whether changes in MCAv from 0° to upright observed < 48 h of stroke modified over time (comparing to 3–7 days post-stroke). Given the limited data on the relationship between early response to upright positions and later functional outcome, we also explored the association between changes in MCAv from lying flat (0°) to upright in stroke participants < 48 h and 30-day functional outcome (modified Rankin Scale, mRS). Finally, we described how physiological measures [systolic blood pressure (BP), diastolic BP, heart rate (HR), and oxygen saturation] changed with head positions.

## 2 Methods

### 2.1 Study design and participants

This was a single-center, prospective cohort study of participants with acute ischemic stroke (with and without occlusive disease) presenting to a stroke unit (Austin Hospital, Melbourne, Australia) and healthy controls.

Eligible stroke participants were >18 years old, presenting with a first or recurrent ischemic stroke, and admitted within 48 h of the onset of symptoms. The exclusion criteria were malignant MCA stroke, (15) significant premorbid disability (mRS > 3), autonomic neuropathy or neurodegenerative disorders, people unable to lie flat, pregnancy, any serious co-morbid illness, and inadequate acoustic temporal windows on TCD. Participants with strokes coming to the stroke unit were screed. Participants or the person responsible were approached, and information on the study was provided by the researchers. Written informed consent was provided by participants or the person responsible if the participant was unable to consent. Participants with aphasia were also included.

We report this cohort study in accordance with Strengthening the Reporting of Observational Studies in Epidemiology (STROBE) guidelines (16). Ethics approval was obtained from the Austin Health Ethics Committee (HREC/15/Austin/34). The study protocol was registered online (ACTRN12615001159549).

### 2.2 Study assessments and procedures

Eligible participants were identified by screening the stroke ward lists. Acoustic temporal window status was already known before obtaining informed consent since neurovascular ultrasounds (carotid Doppler and TCD) were standard tests for patients with acute ischemic stroke at Austin Hospital. Community-living healthy controls were invited to participate via our research network.

Demographic data and stroke-related information included admission stroke severity (NIHSS), clinical stroke subtype using the Oxfordshire Community Stroke Project classification, stroke location, and treatment with tissue plasminogen activator (tPA) or endovascular clot retrieval. Routine acute neuroimaging (CT, MRI) was used to confirm stroke and define stroke location. Within usual care, available acute neuroimaging at admission (CT angiography, CT perfusion, or magnetic resonance angiography) was used to screen for occlusive disease. The persistence of occlusive disease, defined as ≥70% stenosis or occlusion of intra or extracranial arteries supplying the stroke territory, was then confirmed clinically with routine neurovascular ultrasound examination (17, 18).

**Figure 1:**
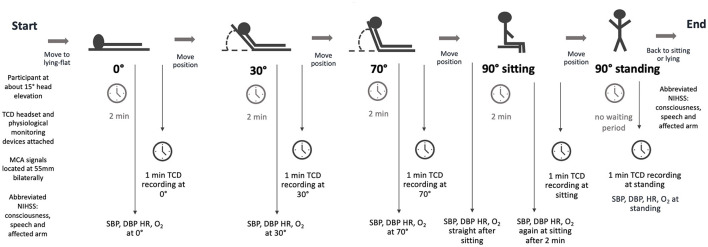
The TCD study protocol. This protocol was performed within 48h of stroke onset, again between 3 and 7 days for participants with stroke, and once for controls. TCD, transcranial Doppler ultrasound; MCA, middle cerebral artery; NIHSS, National Institutes of Health Stroke Scale; SBP, systolic blood pressure; DBP, diastolic blood pressure; HR, heart rate; O_2_, Oxygen saturation.

Accredited neurosonographers (Australian Sonographers Accreditation Registry) with >10 years' experience and a medical doctor (LBC) who received 3 months of training before the commencement of the study performed the TCD head positioning protocol in the neurovascular laboratory adjacent to the stroke unit. The protocol required measurements at five head positions: 0°, 30°, 70°, 90° sitting, and 90° standing (Figure 1). While the primary aim of this study was to determine changes in MCAv between lying flat (0°) and upright positions (90° sitting and 90° standing), we included two intermediate positions (30°, 70°) to allow comparison with previous studies (2). Upright positions incorporating trunk and leg muscle activity were essential to exploring changes related to activities common to early mobilization (i.e., sitting, standing, and walking).

The protocol started with the head of the bed at approximately 15°. The headset, incorporating bilateral ultrasound probes, was attached. MCA signals (peak systolic and end-diastolic velocities) were located bilaterally at a depth of 55 mm using a Compumedics DWL Doppler Box digital TCD device. The bed was then moved to a flat position (0°). After 2 min of acclimatization at 0°, MCA signals were recorded for at least 1 min. This procedure was repeated through 30° and 70° before the participants moved to a sitting position on the bed with feet on the floor without back support. Researchers supported those with compromised trunk control if necessary. To reiterate, 2 min of acclimatization was provided before MCA signals were recorded. Finally, participants stood up with assistance from 1–2 researchers if required. TCD recording started instantaneously after standing up. Physiological measurements (systolic BP, diastolic BP, HR, and oxygen saturation) were also recorded at all time points. The entire procedure took approximately 40 min.

Systolic BP, diastolic BP, and HR were measured using an Omron blood pressure device, and oxygen saturation was measured using an oximetry device. Stroke symptoms and consciousness were also monitored throughout the procedure. New or worsening stroke symptoms or changes in the level of consciousness would result in the termination of testing, and participants would return to a lying-flat position. In addition, three items of the NIHSS scale (consciousness, speech, and affected arm) were assessed immediately before starting the protocol and immediately after to monitor higher cortical and motor function status.

For participants with stroke, the TCD protocol was performed twice: < 48 h after the stroke onset (TCD 1) and between 3 and 7 days after the stroke onset (TCD 2). If TCD 2 could not be performed before hospital discharge, participants were invited to return to complete the study. The TCD protocol was performed once in controls.

### 2.3 Blinded outcome assessment

Three 6-second epochs of the 1-min MCA spectral Doppler recorded in each head position (3 samples per position) were taken to account for variability across the respiratory cycle. All 6-second spectral Doppler images were saved offline, the order was randomized, and the images were de-identified. A neurologist experienced in TCD, blinded to the participant, hemisphere, and head position, determined peak systolic velocity (PSV) and end-diastolic velocity (EDV) for each 6-second epoch. The MCAv for each 6-second epoch was calculated using the formula: MCAv = (PSV-EDV)/3 + EDV (19). Finally, the 3 MCAv values calculated from the 6-second epochs per head position were averaged.

### 2.4 Functional outcome

At 30 days post-stroke, a trained assessor (LBC) conducted a phone interview to collect data on the modified Rankin Scale (mRS), which measures functional disability (20).

### 2.5 Statistical analyses

A power analysis was conducted for the primary hypothesis, which proposed that the change in MCAv from lying-flat to upright positions would differ between participants with or without occlusive disease. Previous systematic review evidence (21), indicated that the difference in MCAv between 0° and 30° in the affected hemisphere of participants with occlusive disease was 8.3 cm/s [95% CI 5.3–11.3; estimated SD = 11.32]. In those without occlusive disease, the differences between 0° and 30° were highly variable and often negligible. Thus, we conservatively assumed that the difference between 0° and 90° positions in patients with occlusive disease would be at least 8.3 cm/s, while no positional change in MCAv would be observed in participants without occlusive disease. Recruiting 62 participants (31 with and without occlusive disease each) would provide 80% power to detect the hypothesized difference, using a 5% two-sided threshold for Type 1 error control.

We measured differences in MCAv from 0° to other positions (30°, 70°, 90° sitting, and 90° standing) in each hemisphere for each group using mixed-effect linear regression modeling. Head position and hemisphere (affected or unaffected, occluded or non-occluded) were used as independent variables, MCAv was the dependent variable, and individual participants were random effects. To determine if differences in the change in MCAv from 0° to upright positions were present between stroke participants with and without occlusive disease (primary hypothesis) and between stroke participants and controls, we investigated position-by-hemisphere interactions by including relevant multiplicative terms in the regression models. To investigate whether changes in MCAv in stroke participants remained consistent over time (secondary aim), we compared changes between both time points: TCD 1 (< 48 h post-stroke) and TCD 2 (3–7 days post-stroke) using the same approach. Position-by-time interactions were investigated by including relevant multiplicative terms in the regression models. These analyses were adjusted for the participant's age, sex, hemisphere, and mean blood pressure at the corresponding position. These variables were chosen based on evidence that MCAv declines with age (22) and that women have approximately 3%−5% higher MCAv than men (23). We also adjusted for the hemisphere to account for within-subject differences in MCAv between hemispheres and for systemic BP at each position to control for potential reductions in blood pressure associated with upright positions that could influence cerebral hemodynamics. Relevant magnitudes of associations were expressed by coefficients of mean differences in MCAv with respective 95% confidence intervals (CI). Missing MCAv data were assumed to be missing at random and were imputed as a part of mixed-effect modeling.

The association between changes in mean MCAv from 0° to upright positions < 48 h of stroke and favorable outcome (mRS 0–2, no or little disability) in participants with stroke was explored using logistic regression analysis, adjusted for age and stroke severity (NIHSS) at admission. The magnitude of the association was expressed as odds ratios (OR) with respective 95% CIs.

The exploration of changes in various physiological measures due to the head position in controls and < 48 h in all stroke participants was conducted using mixed effect modeling, as per the primary analysis.

All analyses were discussed with an experienced statistician (LC). We used Stata 15 IC statistical software (Stata Corp., College Station, TX, USA) to perform all analyses. For the primary hypothesis, *p*-values of < 0.05 were considered statistically significant. Due to the exploratory nature of other hypotheses, we only report effect estimates with respective 95% CIs.

## 3 Results

**Figure 2:**
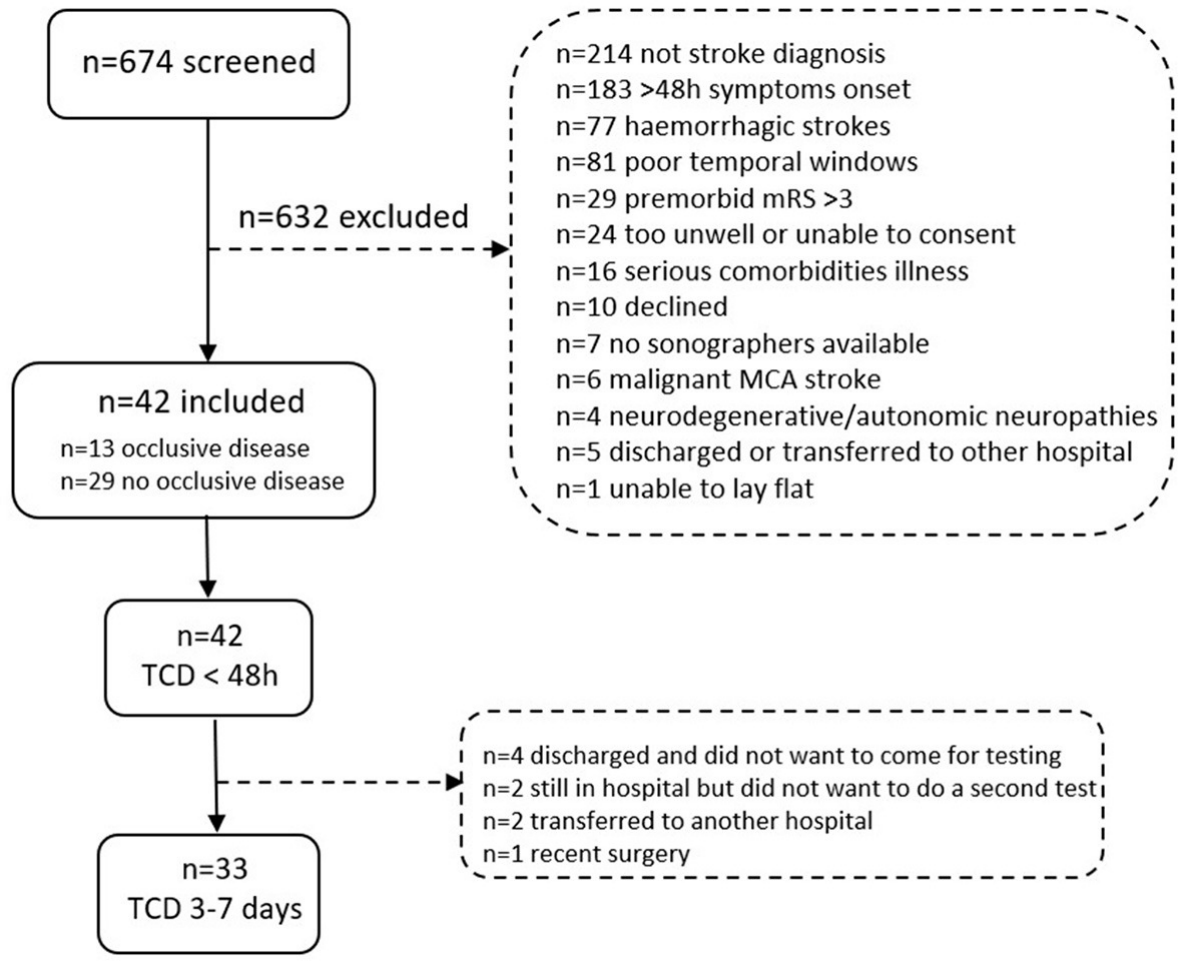
Flow chart with inclusions and exclusions of people with stroke screened at the stroke unit at the Austin Hospital in Melbourne, Australia. Note that some individuals were excluded for more than one reason.

A total of 674 patients were screened, with 52 patients meeting the eligibility criteria and being invited to participate. Of these, 10 patients declined, leaving 42 stroke participants to be included in the study (Figure 2). We ceased recruitment during the COVID-19 pandemic and could not resume, leading to the closure of recruitment in March 2021. A total of 23 controls were recruited, with 22 included and one control excluded due to inadequate temporal acoustic windows. Of the 42 stroke participants in the study, 13 participants were diagnosed with persistent occlusive disease via ultrasound assessments. Of these, four participants had large vessel occlusions, six participants had severe stenosis (>70%), and three participants had large vessel occlusions concurrent with severe stenosis. Regarding the location, seven participants had intracranial, four participants had extracranial, and two participants had both intracranial and extracranial occlusive disease. The affected arteries were the internal carotid artery (ICA) (*n* = 4), MCA (*n* = 4), ICA and MCA (*n* = 3), and vertebral artery (*n* = 2).

Demographics and stroke-related information for the 42 participants with strokes are shown in Table 1. Most participants (62%, *n* = 26) had mild strokes (NIHSS < 8), while four participants (9%) had severe strokes (NIHSS > 16), all of whom were without occlusive disease.

**Table 1:** The characteristics, premorbidity, and stroke-related history of all participants with ischemic stroke were then broken down into those without occlusive disease, those with occlusive disease, and controls.

**Characteristics and risk factors**	**Ischemic stroke *n =* 42**	***No occlusive disease n = 29***	***Occlusive disease n = 13***	**Control *n =* 22**
Age (years), median (IQR)	67.5 (59–77)	61 (59–76)	75 (70–83)	61 (44–69)
Sex Female	13 (31%)	10 (34%)	3 (23%)	13 (59%)
**Risk factors**				
Hypertension	24 (57%)	15 (52%)	9 (69%)	6 (27%)
Diabetes mellitus	8 (19%)	6 (21%)	2 (15%)	1 (5%)
Atrial fibrillation	6 (14%)	5 (17%)	1 (8%)	1 (5%)
Ischemic Heart Disease	6 (14%)	4 (14%)	2 (15%)	1 (5%)
Hypercholesterolemia	14 (33%)	7 (24%)	7 (54%)	4 (18%)
Smoker or ex-smoker^#^	20 (48%)	14 (49%)	6 (46%)	3 (14%)
**Premorbid history**				
Premorbid mRS 0	33 (79%)	24 (83%)	9 (69%)	22 (100%)
1	2 (5%)	2 (7%)		
2	1 (2%)	1 (3%)		
3	6 (14%)	2 (7%)	4 (31%)	
**Stroke history**				
First stroke	31 (74%)	23 (79%)	8 (62%)	
NIHSS at admission, median (IQR)	4 (2–10)	5 (2–13)	2 (2–6)	
Mild (1–7)	26 (62%)	16 (55%)	10 (77%)	
Moderate (8–16)	12 (29%)	9 (31%)	3 (13%)	
Severe (>16)	4 (9%)	4 (14%)		
**Stroke subtype (OCSP classification)**				
Total anterior circulation infarct	8 (19%)	6 (21%)	2 (15%)	
Partial anterior circulation infarct	22 (53%)	15 (52%)	7 (54%)	
Posterior circulation infarct	6 (14%)	3 (10%)	3 (23%)	
Lacunar infarct	6 (14%)	5 (17%)	1 (8%)	
**Stroke affected hemisphere**				
Left	18 (43%)	15 (52%)	3 (23%)	
Cerebral circulation affected				
Anterior	37 (88%)	26 (90%)	11 (85%)	
Posterior	5 (12%)	3 (10%)	2 (15%)	
rtPA treatment^##^	16 (38%)	11 (38%)	5 (38%)	
Endovascular clot retrieval^##^	18 (43%)	14 (48%)	4 (31%)	

^#^Smoker, current smoking or ceased in the last 2 years. Ex-smoker, ceased >2 years. ^##^Treatment with rtPA does not exclude endovascular clot retrieval or vice versa. mRS, modified Rankin Scale; NIHSS, National Institutes of Health Stroke Scale; OCSP, Oxfordshire Community Stroke Project, rtPA, recombinant tissue plasminogen activator.

### 3.1 Missing data and adverse events

All stroke participants completed TCD 1 (< 48 h, median 31.7 h [IQR 26–41]), but only 33 participants completed TCD 2 (3–7 days, median 4 days [IQR 3–6]). Figure 2 shows the reasons for non-completion. For TCD 1, four stroke participants could sit but not stand. While headset stability was maintained for most participants, TCD 2 lost the MCA signal bilaterally in one participant and on the unaffected side in another when standing up. During the testing of one control, the left MCA signal was lost when sitting and standing. For TCD 1, one participant with a stroke experienced back discomfort when moving to the sitting position, and no further TCD or physiological recordings were performed. There were no other adverse events during the TCD protocol, and no changes in NIHSS were observed.

One participant (without occlusive disease) experienced worsening stroke symptoms 8 h after finishing TCD 1 and underwent a carotid stent procedure with some improvement in symptoms. This patient had < 70% stenosis, and it was later found that a mobile component of plaque in the affected vessel was causing the symptoms. An independent medical review deemed this serious adverse event unlikely to be related to the protocol since the patient showed no symptoms during the head positioning study protocol, and symptoms started many hours after the protocol was finished.

One stroke participant who was transferred to another hospital did not complete the 30-day mRS assessment. All remaining participants with stroke (*n* = 41) agreed to the assessment.

### 3.2 Static changes in MCAv and position-by-hemisphere interactions

**Figure 3:**
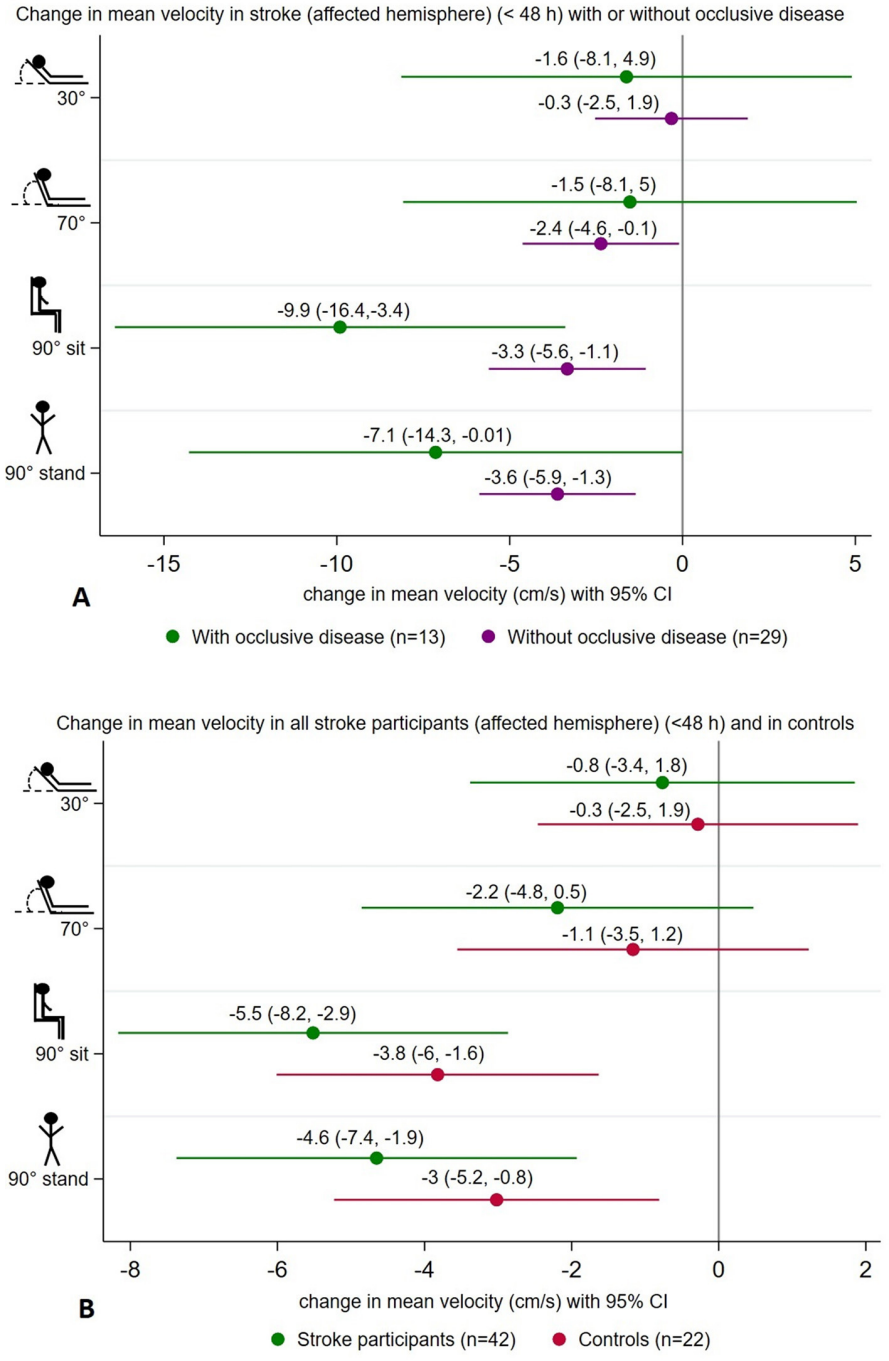
Summary of changes in mean velocity (cm/s) at 30°, 70°, 90° sitting, and 90° standing head positions all relative to lying-flat (0°), showing a significant decrease in mean velocity on the affected hemisphere of stroke participants with occlusive disease (*n* = 13) and without (*n* = 29) at upright positions **(A)**. Similar changes were found on the affected hemisphere of all stroke participants (*n* = 42) and in controls (*n* = 22) **(B)**.

We found a significant decrease in MCAv in the affected hemisphere of stroke participants (< 48 h of stroke onset) with occlusive disease (*n* = 13) from 0° to 90° sitting (−9.9 cm/s, 95% CI [−16.4, −3.4]) and from 0° to 90° standing (−7.1 cm/s, 95% CI [−14.3, −0.01]). Similarly, in those without occlusion (*n* = 29), there was a decrease from 0° to 90° sitting (−3.3 cm/s, 95% CI [−5.6, −1.1]) and from 0° to 90° standing (−3.6 cm/s, 95% CI [−5.9 −1.3]) (Figure 3A). However, position-by-hemisphere interactions between changes in MCAv in the affected hemisphere of stroke participants with occlusive disease (*n* = 13) and without occlusive disease (*n* = 29) (primary hypothesis) did not reach statistical significance (*p* = 0.07).

Considering a stroke in the posterior circulation may not lead to hemodynamic changes in the MCA, we performed exploratory *post-hoc* analyses only in those participants with anterior circulation strokes (*n* = 37). Patterns of change were similar to the whole group, with similar magnitude reductions in MCAv when moving from 0° to more upright positions in the affected hemisphere (< 48 h). In stroke participants with occlusive disease (*n* = 11), the decrease in MCAv at 90° sitting was −11.6 cm/s (95% CI [−19.1, −4.1]), and at 90° standing was −8.9 cm/s (95% CI [−17.3, −0.6]). In those without occlusive disease (*n* = 26), the decrease in MCAv at 90° sitting was −2.8 cm/s (95% CI [−5.2, −0.3]), and at 90° standing was −3.6 cm/s (95% CI [−6, −1.2]). We found a significant interaction between those with occlusive disease (*n* = 11) and those without occlusive disease (*n* = 26) in this subsample (*p* = 0.02). However, the sample size was small, and these findings should be viewed with caution.

Overall, in all stroke participants (< 48 h of stroke onset) (*n* = 42), changes in MCAv in the affected hemisphere differed from 0° to 90° sitting (−5.5 cm/s, 95% CI [−8.2, −2.9]) and from 0° to 90° standing (−4.6 cm/s, 95% CI [−7.4, −1.9]). In controls (*n* = 42), a similar pattern was observed with lower MCAv at 90° sitting (−3.8 cm/s, 95% CI [−6, −1.6]) and at 90° standing (−3 cm/s, 95% CI [−5.2, −0.8]) (Figure 3B). However, no significant position-by-hemisphere interactions were found between stroke participants (*n* = 42) and controls (*n* = 22) (*p* = 0.85). Additionally, we did not find significant differences in MCAv from lying flat to upright positions when comparing the occluded hemisphere (stroke with occlusive disease) and controls (*p* = 0.1), nor when comparing the affected hemisphere in stroke without occlusion and controls (*p* = 0.09).

Table 2 summarizes changes in MCAv from lying-flat (0°) to all other head positions in affected and unaffected hemispheres in stroke participants, with or without occlusive disease at TCD 1 (< 48 h stroke). Table 3 summarizes similar data for TCD 2 (3–7 days) and Table 4 for controls.

**Table 2:** Changes in MCA mean velocities from lying-flat (0°) to other head positions in each hemisphere in stroke participants with occlusive disease or without, <48 h post-stroke (TCD 1).

**Stroke with occlusive disease (*****n*** **=** **13) at TCD 1**				**Stroke without occlusive disease (*****n*** **=** **29) at TCD 1**			
	**Affected hemisphere**	**Unaffected hemisphere**	***p*** **-value for interaction**		**Affected hemisphere**	**Unaffected hemisphere**	***p*** **-value for interaction**
0° to 30°	−1.6 (−8.1,4.9)	2 (−0.6,4.7)	0.97	0° to 30°	−0.3 (−2.5,1.9)	−1.5 (−3.9,0.9)	0.78
0° to 70°	−1.5 (−8.1,5)	1.4 (−1.2,4.1)		0° to 70°	−2.4 (−4.6,−0.1)	−4.9 (−7.4,−2.5)	
0° to 90° sitting	−9.9 (−16.4,−3.4)	−3.8 (−6.5,−1.1)		0° to 90° sitting	−3.3 (−5.6,−1.1)	−4.5 (−7,−2.1)	
0° to 90° standing	−7.1 (−14.3,−0.01)	−3.2 (−6.2,−0.3)		0° to 90° standing	−3.6 (−5.9,−1.3)	−3.9 (−6.3,−1.4)	

Data are expressed as changes in MCA mean velocity (cm/s) with respective 95% confidence intervals. For participants with strokes, TCD 1 was performed < 48 h post-stroke.

**Table 3:** Changes in MCA mean velocities from lying-flat (0°) to other head positions in each hemisphere in stroke participants with occlusive disease or without, 3–7 days post-stroke (TCD 2).

**Stroke with occlusive disease (*****n*** **=** **11) at TCD 2**				**Stroke without occlusive disease (*****n*** **=** **22) at TCD 2**			
	**Affected hemisphere**	**Unaffected hemisphere**	***p*** **-value for interaction**		**Affected hemisphere**	**Unaffected hemisphere**	***p-*** **value for interaction**
0° to 30°	−2.4 (−4.9,0.1)	−1 (−4.3,2.3)	0.9	0° to 30°	−1.3 (−4.3,1.6)	0.6 (−3.7,4.9)	0.43
0° to 70°	−3.2 (−5.7,−0.6)	−3.4 (−6.7,−0.6)		0° to 70°	−3.7 (−6.7,−0.5)	−2.4 (−6.8,2)	
0° to 90° sitting	−5.8 (−8.3,−3.2)	−5.1 (−8.5,−1.8)		0° to 90° sitting	−5.2 (−8.2,−2.14)	−1.3 (−5.8,3.2)	
0° to 90° standing	−7.4 (−9.9,−4.8)	−3.3 (−6.6,−0.3)		0° to 90° standing	−6.7 (−9.7,−3.6)	−0.9 (−5.4,3.5)	

Data are expressed as changes in MCA mean velocity (cm/s) with respective 95% confidence intervals. For participants with strokes, TCD 2 was performed 3–7 days post-stroke.

**Table 4:** Changes in MCA mean velocities from lying-flat (0°) to other head positions in each hemisphere in controls.

**Controls (*****n*** **=** **22)**			
	**Left hemisphere**	**Right hemisphere**	***p*** **-value for interaction**
0° to 30°	0.3 (−2,2.6)	−0.8 (−2.8,1.2)	0.76
0° to 70°	−0.2 (−2.6,2.3)	−2 (−4.2,0.2)	
0° to 90° sitting	−3.5 (−5.9,−1.2)	−3.5 (−5.5,−1.6)	
0° to 90° standing	−1.9 (−4.3,0.4)	−3.5 (−5.5,−1.5)	

Data are expressed as changes in MCA mean velocity (cm/s) with respective 95% confidence intervals.

### 3.3 Position-by-time interaction (TCD 1 and TCD 2)

We found no differences in static changes in MCAv over different positions between TCD 1 (< 48 h) (*n* = 42) and TCD 2 (3–7 days) (*n* = 33) (*p* = 0.64).

### 3.4 Static changes in MCAv and 30-day-mRS

Of the 41 participants with stroke who were followed up at 30 days, 29 participants had favorable outcomes (mRS 0–2). Of those without occlusive disease, 78% (*n* = 22/28) had a favorable outcome, compared to only 54% (*n* = 7/13) of those with occlusive disease. In the overall stroke sample (*n* = 41), we found no association between changes in MCAv in TCD 1 (< 48 h) from 0° to 90° sitting (adjusted OR 1.005, 95% CI [0.95,1.06]) or from 0° to 90° standing (adjusted OR 1.01, 95% CI [0.91,1.11]) and a favorable outcome.

### 3.5 Physiological assessments

**Figure 4:**
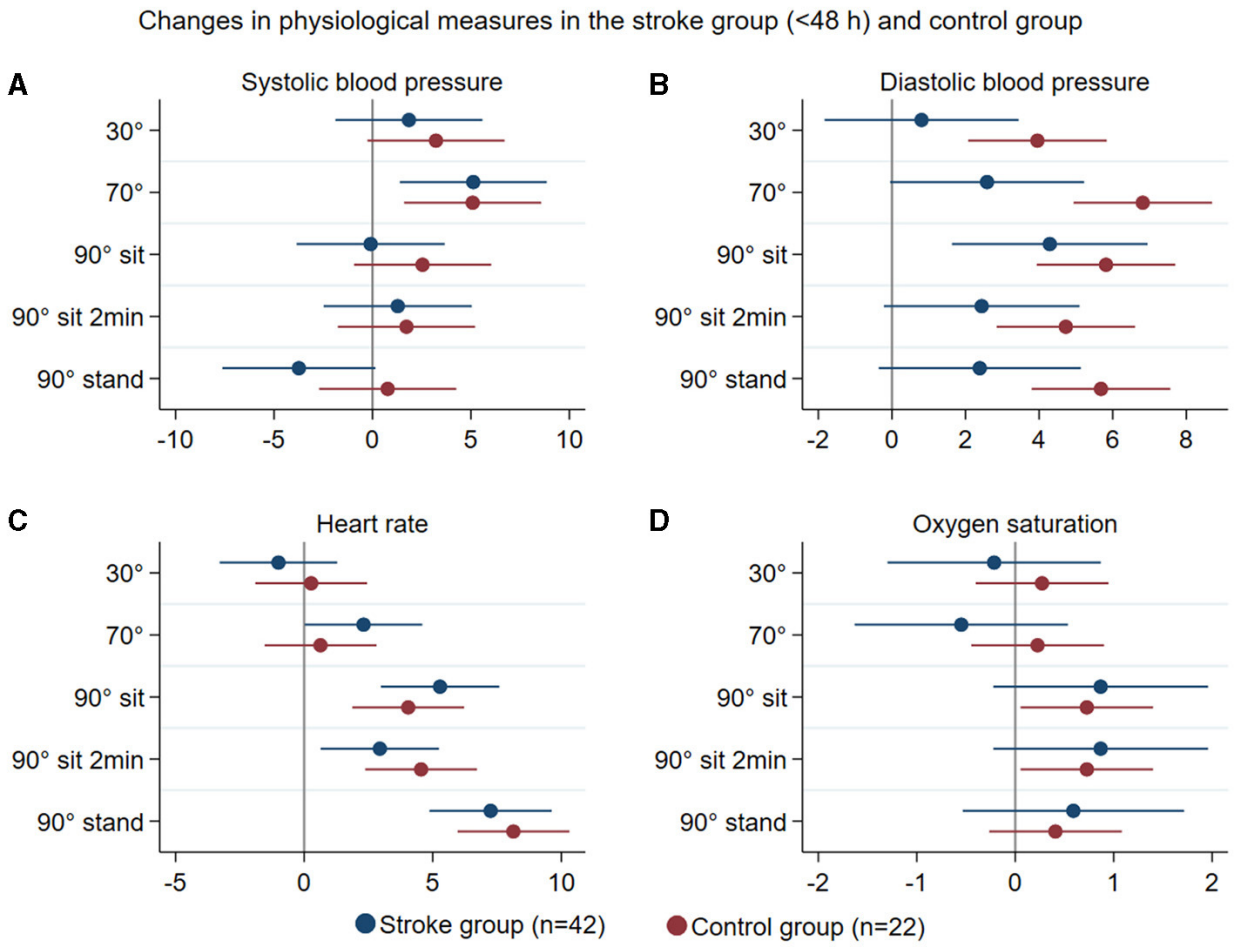
Summary of mean changes in systolic blood pressure (mmHg) **(A)**, diastolic blood pressure **(B)**, heart rate **(C)**, and oxygen saturation **(D)** in all stroke participants (blue) and controls (brown) at different head positions, all in relation to 0°.

**Figure 5:**
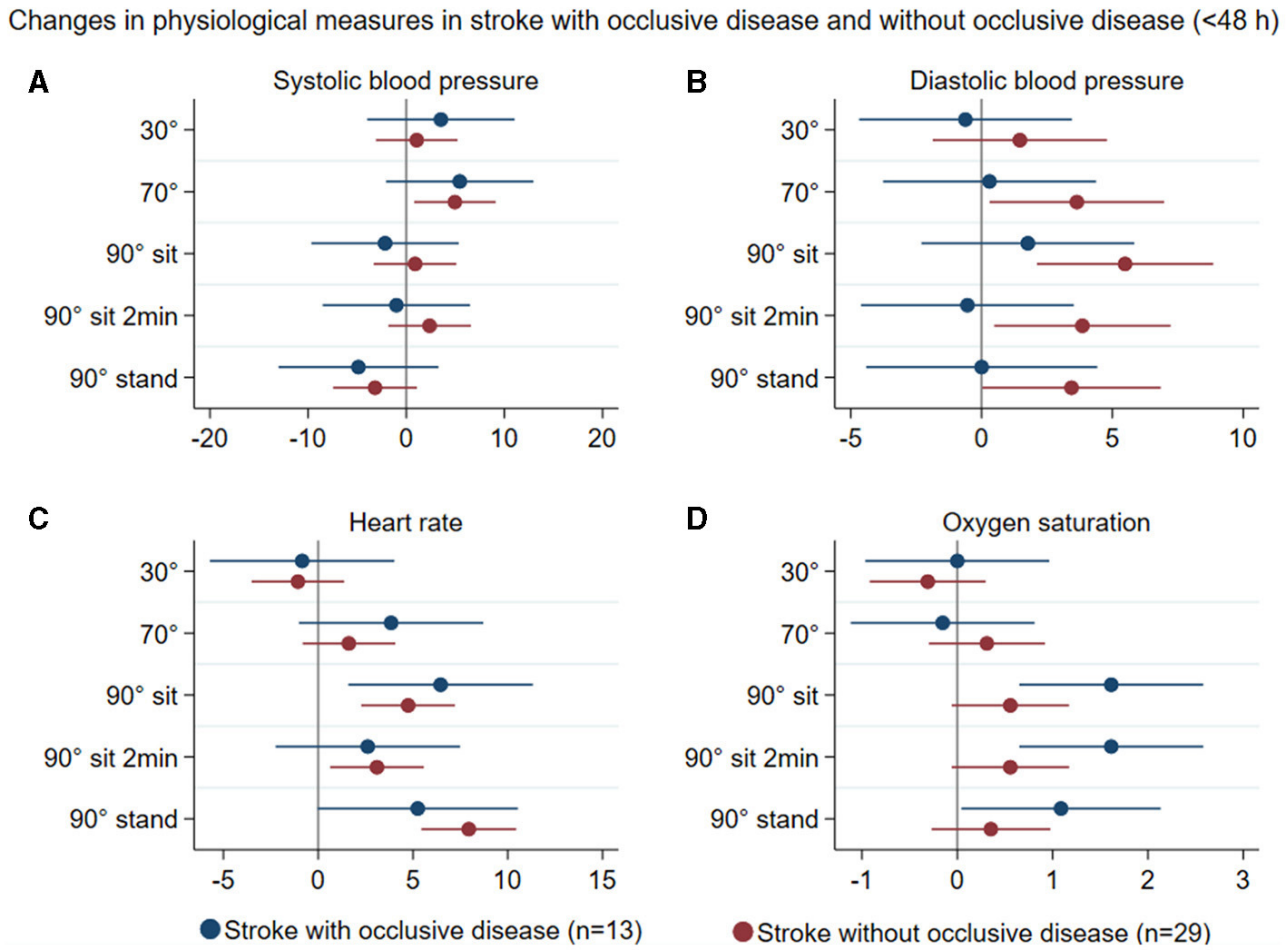
Summary of mean changes in systolic blood pressure (mmHg) **(A)**, diastolic blood pressure **(B)**, heart rate **(C)**, and oxygen saturation **(D)** in stroke participants with occlusive disease (blue) and stroke participants without occlusive disease (brown) at different head positions, all in relation to 0°.

Patterns of changes in physiological measures (systolic and diastolic blood pressure, heart rate, and oxygen saturation) with position change from 0° to all other head positions in all stroke participants < 48 h (*n* = 42) and in controls (*n* = 22) are represented in Figure 4. In stroke participants with and without occlusive disease, these changes are displayed in Figure 5. There was no significant difference between groups in these variables.

## 4 Discussion

The present study aimed to further explore this unclear relationship between upright positioning, occlusive disease, cerebral hemodynamics, and clinical outcomes in acute ischemic stroke, particularly in people with occlusive disease. While we found a significant decrease in MCAv when moving from lying-flat (0°) to upright 90° sitting and 90° standing in stroke participants (including anterior and posterior circulation strokes), this was not significantly different between those with and without occlusive disease. Additionally, an early decrease in MCAv was not associated with later functional outcomes. However, given that differences in responses between those with and without occlusive disease approached significance, and subgroup analysis of those with anterior circulation stroke only showed a significant reduction in MCAv in stroke with occlusive disease compared to those without, we believe it would be valuable to examine responses of a larger sample of patients with occlusive disease in future studies.

The need for further investigations into the potential mechanisms for poorer outcomes in stroke patients treated with high doses of early out-of-bed upright activity (sitting, standing, and walking) in the AVERT (6) trial, combined with the hypothesized role that upright positioning (sitting and standing) may play in reducing perfusion to the penumbra and worsening outcomes for stroke patients, particularly those with occlusive disease, guided the development of this study. Unfortunately, occlusive disease status was not systematically collected in the AVERT trial. In an exploratory subgroup analysis (one hospital, *n* = 190 patients with acute ischemic stroke) from the AVERT trial, neurovascular ultrasound reports were retrospectively assessed to investigate the presence of occlusive disease. This small study showed less favorable results in stroke patients with occlusive disease (*n* = 36) who started higher dose upright activity early compared to usual care. However, the results were not significant, highlighting the need for further prospective studies to investigate this matter (24).

Interrupted by the pandemic, this study faced a significant limitation, as it was underpowered to definitively test our hypothesis. Nevertheless, given the relatively large sample and acute timing compared to previous studies (2), we believe our results provide valuable insights into the effects of upright postures on cerebral hemodynamics in the early days after a stroke. Due to size limitations, we combined stroke participants with acute large vessel occlusion and longstanding severe stenosis into a single group of participants with persistent occlusive disease. Of those with occlusive disease, patients with intracranial and complete occlusions are a potentially vulnerable group. Given we had only 13 cases in the occlusive disease group, this subgroup may not be well represented in the small sample. Vulnerability would also be particularly high for those with poor collaterals (25–27). Another limitation is that, although we used routine neurovascular ultrasound examination to confirm the presence of occlusive disease just before performing the TCD head positioning protocol, the persistence of occlusive disease could not be confirmed at the exact time when changes in posture were assessed. Additionally, hemodynamic changes in mean velocity may be influenced by factors other than the presence of occlusive disease and could depend particularly on downstream collateral flow. While we did not record collateral status in the current study, it would be valuable to include it in future research. We also acknowledge that the lower NIHSS scores in the occlusive disease group compared to the non-occlusive disease group may raise some concerns about the generalizability of the results. We believe that the small sample size and inclusion of individuals with longstanding severe stenosis may have lowered the overall NIHSS score in the occlusive disease group. Furthermore, since the study was conducted in a highly efficient acute stroke center, only a small percentage of stroke patients remained with persistent large vessel occlusions after thrombolytic therapy, with the most severe and clinically unstable cases being excluded from the study. Future studies should consider strategies to enhance the recruitment of patients with severe strokes and persistent large vessel occlusion.

Importantly, our protocol only tested responses to short intervals of upright positions in a stepwise manner, with slow progressions to sitting and standing. Unlike the AVERT trial, it did not test more frequent upright activity. Whether extended periods of upright positions commonly associated with upright activity, repetitive stimuli such as sit-to-stand or intermittent walking, or more vigorous upright activity can lead to even greater changes in MCAv is unknown. While long static recordings may show no difference in MCAv, more vigorous upright activity could be challenging to the penumbra tissue if dynamic cerebral autoregulation is impaired, especially in a population with more severe strokes than those included in this study. Moreover, we acknowledge that concepts of dynamic cerebral autoregulation, transfer function analysis, and measurement of end-tidal CO_2_ were not considered when the study protocol was conceived. Since there may be changes in ventilation in some patients when moving from supine to upright posture, it is possible that MCA mean velocity could have been affected by a change in pCO_2_ (28). Another limitation is that we assessed static posture changes in this study, allowing blood pressure and MCAv to stabilize before measurements were taken. Beat-to-beat measures of conductance with postural changes would better inform us about the pressure-flow relationship to a challenging stimulus and dynamic cerebral autoregulation. Therefore, such monitoring ought to be included in future research in this field.

While questions remain about the effect of upright positions on hemodynamics, our finding that MCAv between 0° and 30° head positions did not change in any groups suggests that patients with acute ischemic stroke can be nursed in either of these two positions without concern. Our data are consistent with other studies in acute stroke and are in line with results from the large multicentre HeadPoST trial (1), which found no changes in functional outcome (90-day mRS) after nursing patients with acute stroke either in a lying-flat or 30° position for 24 h after randomization.

We chose TCD to assess changes in cerebral hemodynamics for several reasons. Although TCD has some limitations, for instance, measuring velocity rather than flow or perfusion, it remains the optimal technique for continuously monitoring cerebral hemodynamics parameters with changes in the head position, as it allows for real-time bedside assessments of intracranial MCAv (19, 29). This was particularly important for our study as it allowed participants' head and body movements without compromising any recordings. Furthermore, there is support for using TCD as a surrogate for CBF, provided that the diameter of the artery remains stable (30–32). Experienced neurosonographers performed all TCDs, and we only included participants with adequate temporal windows (33). Another strength of the study design was having offline blinded assessments of the TCD spectral Doppler images by an experienced assessor to avoid possible bias in TCD outcomes.

Regarding physiological assessments, stroke participants showed a response pattern to moving upright similar to that of controls. These responses were consistent with expected changes in healthy individuals immediately after standing up: an increase in diastolic BP (< 10 mmHg), little or no change in systolic BP, and an increase in HR of around 10 bpm (34), with the exception that stroke participants showed less consistent changes in BP, particularly in diastolic BP, than controls. People with stroke can present with orthostatic intolerance and experience orthostatic hypotension (35, 36); however, we observed no large drops in BP in our population throughout the study. Our stepwise protocol with a gradual rise to upright positions may have contributed to this finding. The pattern of changes in systemic BP as participants moved to more upright positions was inconsistent with MCAv changes. These findings once again suggest that systemic BP should not be used as a proxy marker for changes in CBF parameters (2).

We originally planned to include patients with both anterior and posterior circulation strokes in this study and explore how a posterior circulation stroke might affect changes in MCAv in the anterior circulation. This has not been addressed in previous cerebral hemodynamics studies, and little is known about the hemodynamic responses of this subgroup. However, we realized early on that we were unlikely to accrue sufficient participants with posterior circulation strokes to address this question. We retained these individuals in the current analysis for completeness. Given that their exclusion did alter the results in our *post hoc* analysis, it is likely that patients with posterior circulation stroke do not respond in the same manner to upright postures and/or that the MCAv is not representative of cerebral blood flow changes with changes in position in this population. Since most TCD headsets are built to monitor the MCAv, monitoring the PCA through the temporal window or the vertebral artery through the occipital window would require more complex techniques to ensure a constant insonation angle during head position changes. We would recommend separate studies of individuals with posterior circulation strokes to better understand their specific hemodynamic responses.

In conclusion, as people sit and stand up from a lying position, MCAv is reduced in most cases in the affected hemisphere, which is true for patients with and without occlusive disease and healthy community-dwelling individuals. The decrease in MCAv when moving from lying to upright was similar in strokes with anterior and posterior circulation strokes with occlusive disease compared to those without. However, subgroup analysis of anterior circulation stroke showed a significant difference in the decrease in MCAv when moving from lying to upright in stroke patients with occlusive disease compared to those without occlusive disease. Considering the important clinical hypothesis that reduced cerebral perfusion may impact the ischemic penumbra during upright activity, more research is warranted, especially in people with acute ischemic stroke with concurrent occlusive disease.

## Data availability statement

The raw data supporting the conclusions of this article will be made available by the authors, without undue reservation.

## Ethics statement

The studies involving humans were approved by the Austin Health Ethics Committee. The studies were conducted in accordance with the local legislation and institutional requirements. The participants provided their written informed consent to participate in this study.

## Author contributions

LBC: Data curation, Formal analysis, Investigation, Methodology, Project administration, Resources, Visualization, Writing – original draft, Writing – review & editing. TK: Formal analysis, Methodology, Writing – review & editing. BC: Conceptualization, Funding acquisition, Methodology, Resources, Supervision, Writing – review & editing. KB: Funding acquisition, Investigation, Methodology, Supervision, Writing – review & editing. CL: Conceptualization, Writing – review & editing. LC: Formal analysis, Writing – review & editing. VT: Investigation, Methodology, Resources, Supervision, Writing – review & editing. JB: Conceptualization, Formal analysis, Funding acquisition, Investigation, Methodology, Resources, Supervision, Writing – review & editing.
